# ONEIDA HELPING ONEIDA: DECISION MAKING ABOUT SERVICE USE BY ONEIDA NATION FAMILY CAREGIVERS

**DOI:** 10.1093/geroni/igad104.0812

**Published:** 2023-12-21

**Authors:** Mary Wyman, Haley Buerger-Cole, Nickolas Lambrou, Joseph Perzynski, Sacheen Lawrence, Debra Miller, Florence Petri, Jordan Lewis

**Affiliations:** Madison VA/Univ Wisconsin, Madison, Wisconsin, United States; University of Wisconsin, Madison, Wisconsin, United States; University of Wisconsin, Madison, Wisconsin, United States; Madison VA Hospital, Madison, Wisconsin, United States; University of Wisconsin, Madison, Wisconsin, United States; Oneida Comprehensive Health, Madison, Wisconsin, United States; Oneida Nation of Wisconsin, Madison, Wisconsin, United States; University of Minnesota Medical School, Duluth campus, Duluth, Minnesota, United States

## Abstract

Native American communities are disproportionately impacted by Alzheimer’s disease and related dementias and are more likely to identify as family caregivers compared to other racial groups. Caregiver resources such as educational, supportive, and respite services have been shown to decrease burden and improve health outcomes. However, government and tribal services to support caregivers are underutilized. Using a CBPR approach, within the framework of an existing partnership between the Oneida Nation of Wisconsin and the Wisconsin Alzheimer’s Disease Research Center, all phases of research were conducted with Oneida partners. We used a mixed-method approach to gain a better understanding of the cultural and community-related factors impacting resource utilization by Native American dementia caregivers. Ten caregivers of older adults with memory loss completed surveys followed by in-depth interviews. Caregivers were aged 38-69 years, 90% female, and living on or near the Oneida reservation; time caregiving varied from <1 to >10 years. Most care recipients had not received a formal diagnosis of memory disorder. Interviews revealed barriers to service use including low awareness, complexities of service funding structures, and care recipient preferences. Perceptions of alignment of traditional cultural values with service use were nuanced and varied. Our findings underline the multiple factors impacting Native families coping with dementia and contribute to the scant literature on ADRD caregiving among Native Americans. Results can inform public health initiatives to address ADRD disparities and improve outcomes for Indigenous persons with ADRD.

